# Multispectral and X-ray images for characterization of *Jatropha curcas* L. seed quality

**DOI:** 10.1186/s13007-021-00709-6

**Published:** 2021-01-26

**Authors:** Vitor de Jesus Martins Bianchini, Gabriel Moura Mascarin, Lúcia Cristina Aparecida Santos Silva, Valter Arthur, Jens Michael Carstensen, Birte Boelt, Clíssia Barboza da Silva

**Affiliations:** 1 Department of Crop Science, College of Agriculture “Luiz de Queiroz”, University of São Paulo, 11 Padua Dias Ave, Box 9, Piracicaba, SP 13418-900 Brazil; 2 Laboratory of Environmental Microbiology, Brazilian Agricultural Research Corporation, Embrapa Environment, Rodovia SP 340, Km 127.5, Jaguariúna, 13820-000 Brazil; 3 Laboratory of Radiobiology and Environment, Center for Nuclear Energy in Agriculture, University of São Paulo, 303 Centenario Ave., Sao Dimas, Piracicaba, SP 13416-000 Brazil; 4 Technical University of Denmark, 2800 Lyngby, Denmark; 5 Department of Agroecology, Science and Technology, Aarhus University, 4200 Slagelse, Denmark

**Keywords:** *Jatropha curcas*, Non-invasive methods, Radiographic images, Artificial intelligence

## Abstract

**Background:**

The use of non-destructive methods with less human interference is of great interest in agricultural industry and crop breeding. Modern imaging technologies enable the automatic visualization of multi-parameter for characterization of biological samples, reducing subjectivity and optimizing the analysis process. Furthermore, the combination of two or more imaging techniques has contributed to discovering new physicochemical tools and interpreting datasets in real time.

**Results:**

We present a new method for automatic characterization of seed quality based on the combination of multispectral and X-ray imaging technologies. We proposed an approach using X-ray images to investigate internal tissues because seed surface profile can be negatively affected, but without reaching important internal regions of seeds. An oilseed plant (*Jatropha curcas*) was used as a model species, which also serves as a multi-purposed crop of economic importance worldwide. Our studies included the application of a normalized canonical discriminant analyses (nCDA) algorithm as a supervised transformation building method to obtain spatial and spectral patterns on different seedlots. We developed classification models using reflectance data and X-ray classes based on linear discriminant analysis (LDA). The classification models, individually or combined, showed high accuracy (> 0.96) using reflectance at 940 nm and X-ray data to predict quality traits such as normal seedlings, abnormal seedlings and dead seeds.

**Conclusions:**

Multispectral and X-ray imaging have a strong relationship with seed physiological performance. Reflectance at 940 nm and X-ray data can efficiently predict seed quality attributes. These techniques can be alternative methods for rapid, efficient, sustainable and non-destructive characterization of seed quality in the future, overcoming the intrinsic subjectivity of the conventional seed quality analysis.

## Background

A crucial step for crop success is the use of high-quality seeds for obtaining vigorous and uniform seedlings. High-quality seeds are more resistant to biotic and abiotic stresses [1], and they originate seedlings that provide rapid soil coverage, using more efficiently the available radiation and nutrients, reducing the potential side-effects caused by weed-crop competition [2].

Currently, quality monitoring operations are estimated mainly by visual inspection of seeds one by one using germination and vigor tests, which are destructive, laborious and requiring trained seed analysts. New and refined technologies based on computerized procedures create the opportunity for automating laboratory evaluations, providing decision-making support regarding the destination of seedlots. In addition, the development of fast, non-invasive and less subjective tools is relevant for seed industry.

Multispectral imaging can provide spatial and spectral information related to different quality traits, such as surface structure, texture and chemical composition [3]. Briefly, this technique is based on sequential exposure of an object to light at different wavelengths integrated with computer systems to recognize physicochemical parameters from biological samples. In the context of seed quality, spectral imaging is mainly based on texture [4], physical and chemical attributes associated with insect damages [5], fungal infections [6], among others, but with limitation to examine internal tissues. Meanwhile, X-ray imaging has proved great potential to collect data from internal structures (e.g. damages in embryo and endosperm) [7, 8], because X-rays are short electromagnetic waves (ranging from 0.01 to 10 nm), with high penetrating power [9]. The correlation of these technologies with data obtained by traditional analytical methods can generate new quality markers [10]. A particular combination of spectral data with X-ray imaging can also improve the performance of classifiers [11], providing complementary information related to morphological and biochemical parameters.

In this study, we tested multispectral and X-ray imaging compared to conventional analytical methods for seed quality characterization. *Jatropha curcas* was used as model species, which has been a focus in the study of plants that can be used to produce biofuel, food, feed and biogas from seed cakes [12–16]. To the best of our knowledge, there have been no attempts in using multispectral imaging combined with radiographic images prior to this study.

## Results

### Seed vigor classification based on traditional tests

Overall, seeds from Lot 2 showed the best performance in the germination tests (Table 1). Lot 1 had the lowest germination rate, and Lot 3 showed an intermediate performance (germination on paper substrate). In the vigor tests, Lot 2 was classified as higher vigor, with the germination rate index–GRI having greater sensibility in separating seedlots based on vigor: low vigor = Lot 1, high vigor = Lot 2, and medium vigor = Lot 3 (Table 2).

**Table 1:** Germination tests to rank *Jatropha curcas* seedlots based on germination capacity (normal seedlings) at 7 and 10 days, using two different substrates (paper and sand)

Seedlot	Germination [%] 5 days		Germination [%] 10 days	
	Paper	Sand	Paper	Sand
1	13 ± 3.00 c^a^	59 ± 5.25 b	18 ± 2.90 c	63 ± 5.97 b
2	87 ± 3.00 a	94 ± 1.15 a	98 ± 1.33 a	97 ± 1.00 a
3	58 ± 5.54 b	70 ± 2.58 b	60 ± 5.58 b	70 ± 2.58 b
ANOVA	F_2,27_ = 85.70 *P* = 0.0000	F_2,9_ = 26.94 *P* = 0.002	F_2,27_ = 116.22 *P* = 0.0000	F_2,9_ = 22.31 *P* = 0.0003

^a^Means (± standard error) within each column followed by the same letter are not significantly different according to Tukey test (*P* < 0.05)

**Table 2:** Vigor tests to rank *Jatropha curcas* seedlots based on vigor: germination rate index, electrical conductivity and seedling emergence at 10 days

Seedlot	Germination rate index	Electrical condutivity [µS cm^−1^ g^−1^]	Seedling emergence [%]
1	0.6 ± 0.49 c	188.8 ± 7.88 b	66 ± 4.76 b^a^
2	4.0 ± 0.06 a	79.1 ± 3.69 a	91 ± 3.00 a
3	2.5 ± 0.17 b	91.8 ± 5.61 a	59 ± 9.29 b
ANOVA	F_2,27_ = 81.31 *P* = 0.0000	F_2,9_ = 100.71 *P* = 0.0000	F_2,9_ = 7.19 *P* = 0.0136

^a^Means (± standard error) within each column followed by the same letter are not significantly different according to Tukey test (*P* < 0.05)

**Fig. 1:**
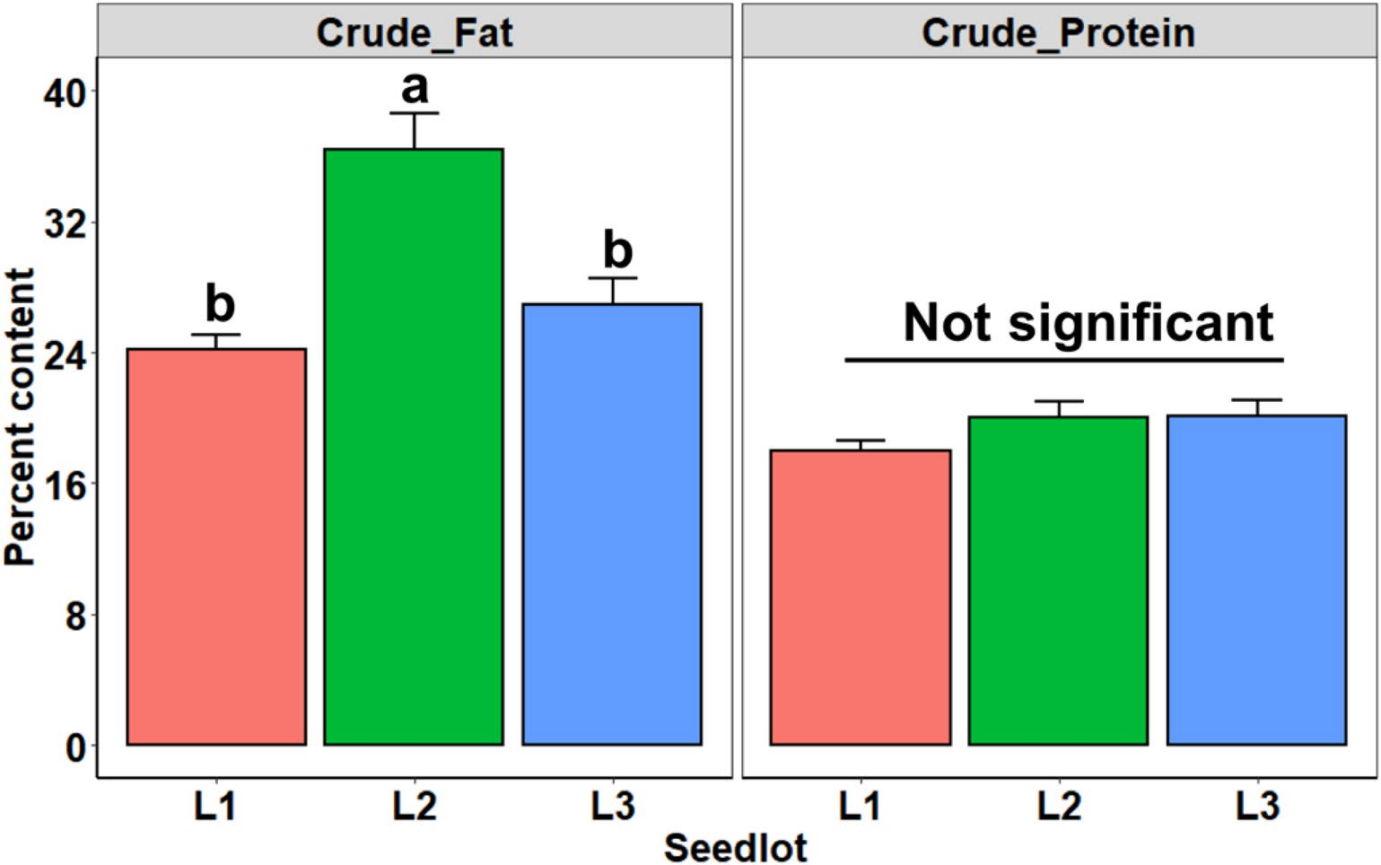
Crude fat and crude protein content of three seedlots of *Jatropha curcas* categorized by different vigor status (low vigor: = Lot 1, high vigor = Lot 2, and medium vigor = Lot 3). ANOVA was performed separately for each variable, and different letters indicate significant contrasts between means (bars + SE, upper standard error) according to Tukey HSD test (*P* < 0.05)

Crude fat content greatly varied among seedlots (*F* = 15.37, df = 2, 6, *P* = 0.004), with Lot 2 exhibiting 51% and 35% more fat than Lot 1 and Lot 3, respectively (Fig. 1). Conversely, the crude protein content, known to have a marked effect on the rapid imbibition of water by seeds, did not significantly differ among the seedlots (*F* = 2.03, df = 2, 6, *P* = 0.212) (Fig. 1).

### Multispectral imaging integrated with X-ray imaging

**Fig. 2:**
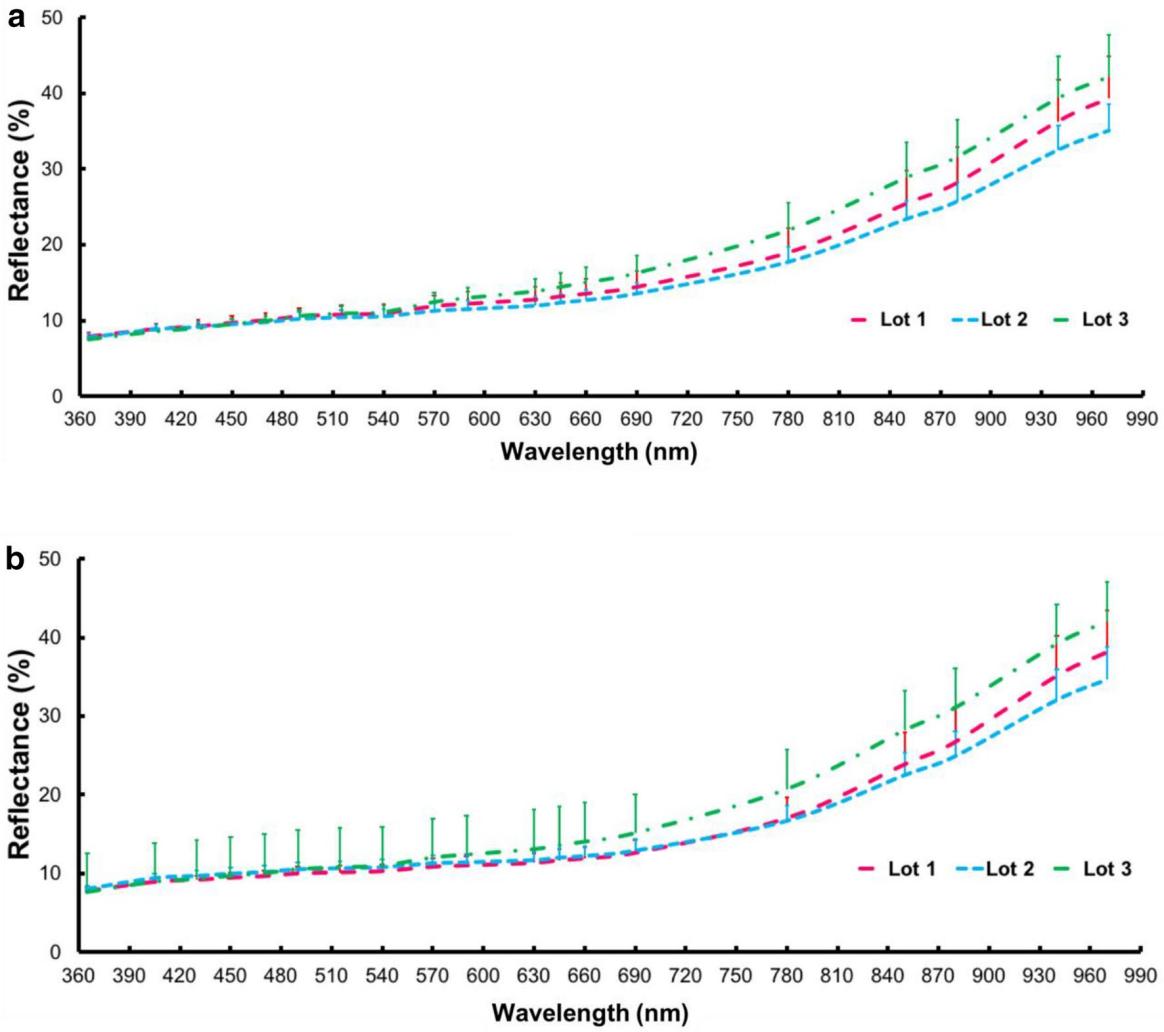
Plot of mean spectrum within each region of interest–ROI (n = 100 seeds per lot) at 19 wavelengths for ventral (**a**) and dorsal (**b**) seed surface of three lots of *Jatropha curcas* with different vigor levels: low vigor = Lot 1, high vigor = Lot 2, and medium vigor = Lot 3. Vertical bars represent standard deviation (in upper directions)

Seed orientation was not relevant to discriminate seedlots based on multispectral reflectance (Fig. 2). The wavelengths from ultraviolet (365 nm) and visible (405–690 nm) regions showed low reflectance intensity (< 20%) and it was difficult to distinguish the lots. However, data obtained at longer wavelengths, particularly in the near infrared (NIR) region (from 780 to 970 nm) clearly enabled discrimination among seedlots, and seeds with high vigor showed the lowest reflectance intensity (Lot 2).

**Fig. 3:**
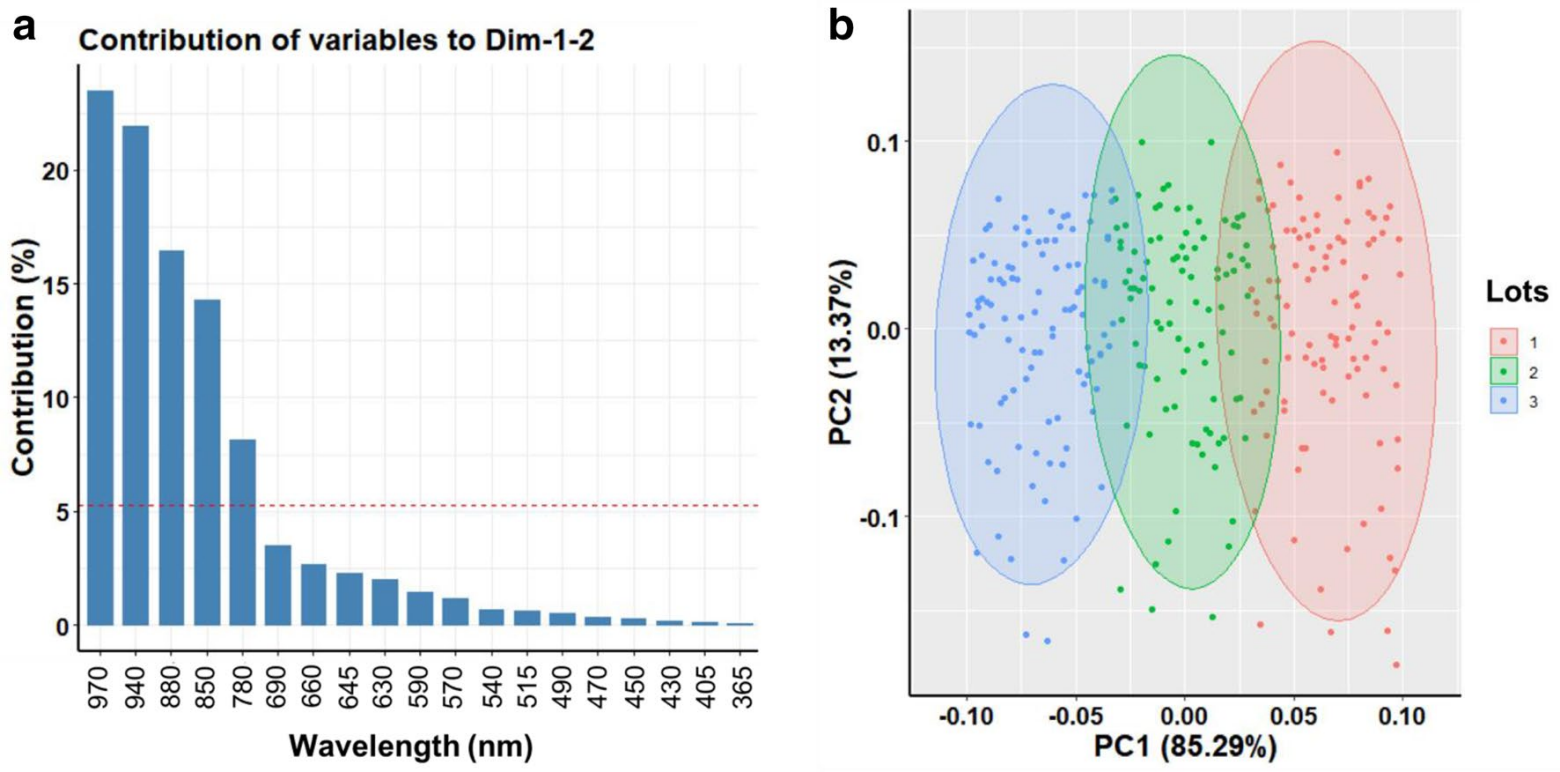
**a** Contributions of response variables to dimensions 1 and 2 with red-dashed line indicating the cut-off for significant variables according to Pearson's correlation test (*P* < 0.05). **b** Biplots of principal component analysis (PCA) for multispectral reflectance in *Jatropha curcas* seedlots (Lots 1, 2 and 3). The five wavelengths from 780 to 970 nm significantly explained the differences among seedlots (*F* = 32.22, df = 10, 586, *P* < 0.0001)

A principal component analysis (PCA) was applied to the multispectral data to reduce variables and it revealed the first two principal components accounted for most of the data variability (98.66% of total data variance) among seedlots. The contribution histogram indicated the most informative wavelengths based on 95% confidence threshold (cut-off) represented by the dashed red line, which revealed only five bandwidths from 780 to 970 nm that mostly contributed to PC1 and PC2 (Fig. 3a). Cluster analysis based on PC1 and PC2 distinctly separated the three seedlots (Fig. 3b).

**Fig. 4:**
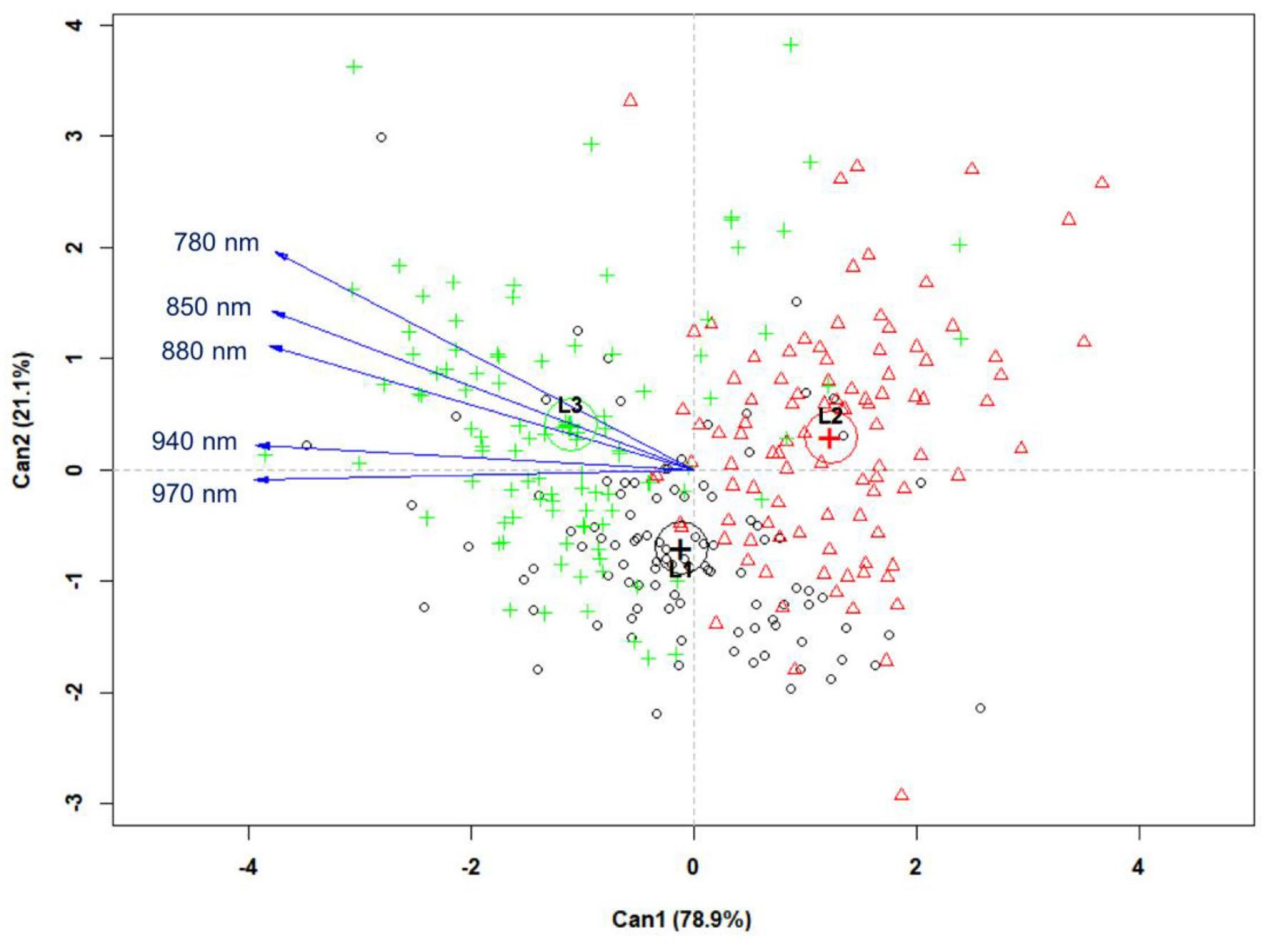
Canonical discriminant analysis (CDA) of reflectance data obtained at 780, 850, 880, 940 and 970 nm from three seedlots of *Jatropha curcas* with different vigor levels: low vigor = L1 [black circles], high vigor = L2 [red triangles], and medium vigor = L3 [green crosses]) (n = 100 seeds per lot)

In order to validate the PCA model, multispectral data corresponding to the five most important wavelengths (780, 850, 880, 940 and 970 nm), as previously assigned by PCA were used in a canonical discriminant analysis (CDA) (Fig. 4). Lots 1 and 3 had lower vigor, and those wavelengths positively distinguished Lots 1 and 3 from Lot 2 (high vigor), which suggests that these wavelengths are good predictors for discriminating jatropha seedlots with lower vigor.

**Fig. 5:**
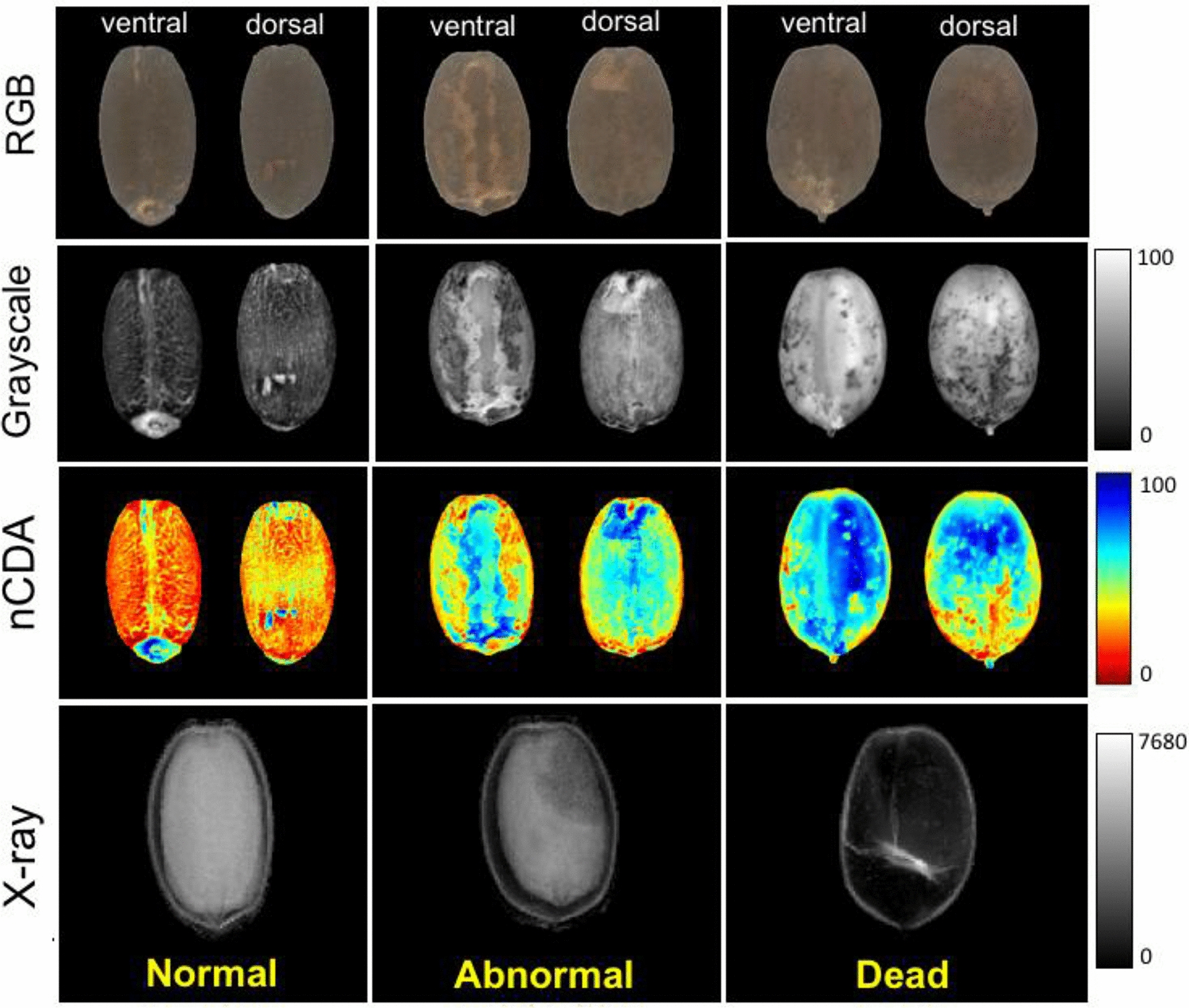
Raw RGB images of ventral and dorsal surfaces of *Jatropha curcas* seeds based on germination capacity and corresponding reflectance images captured at 940 nm (grayscale and transformed images using nCDA algorithm) and X-ray images. RGB images are represented by three-color channels (red, green and blue) to generate a single-color value for each pixel in the image. In the grayscale and transformed images using nCDA each pixel is represented by a single-value that correspond to reflectance intensity. Higher pixel values in the X-ray images indicate higher tissue density

Jatropha seed has a thick and dark tegument. Figure 5 shows RGB images obtained from ventral and dorsal surface of the seeds based on germination capacity and corresponding reflectance images captured at 940 nm and X-ray images. Since the CDA model showed collinearity between 940 and 970 nm (Fig. 4), we selected the reflectance images captured at 940 nm (Fig. 5) because this wavelength is strongly associated with absorbance peak of fatty tissues, which is relevant for oilseed studies.

In the germination test, normal seedlings were produced from seeds with lower pixel values in the reflectance images and higher pixel values in the X-ray images. There was a different pattern from those seeds that generated abnormal seedlings or did not germinate (dead seeds) in which dead seeds exhibited the highest and lowest pixel values in the reflectance and X-ray images, respectively.

**Fig. 6:**
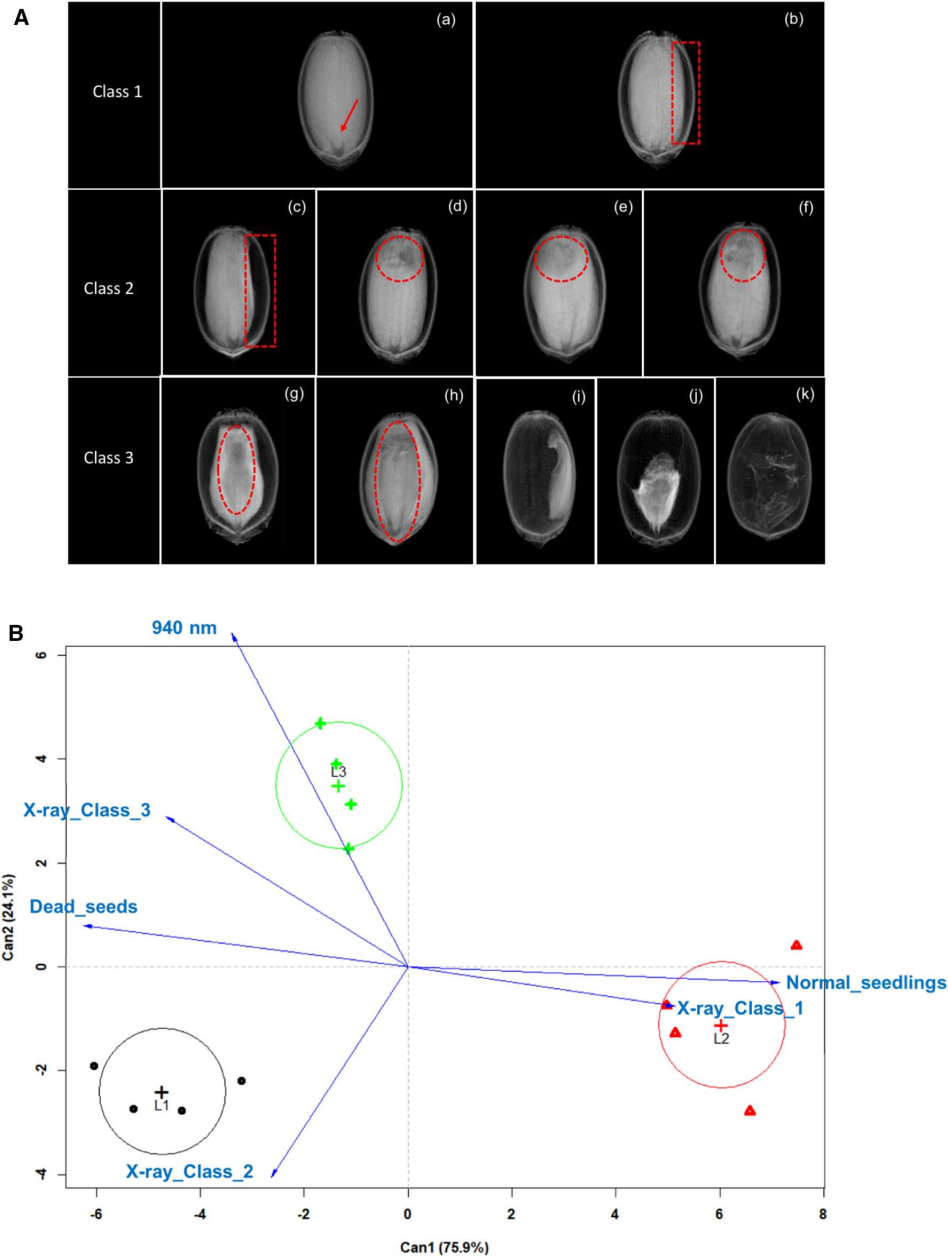
**A** X-ray images of *Jatropha curcas* seeds separated into three different classes based on tissue integrity: Class 1–(**a**) tissues completely filling the seed [arrow indicates the embryonic axis region], (**b**) slight empty spaces (≤ 1.23 mm) between the endosperm and the seed coat; Class 2–(**c**) large empty spaces (> 1.24 mm) between the endosperm and the seed coat; (**d**–**f**) deteriorated tissues without reaching the embryonic axis; Class 3–(**g**, **h**) deteriorated tissues reaching the embryonic axis, (**i**, **j**) malformed seeds, (**k**) empty seed. **B** Canonical discriminant analysis (CDA) of X-ray classes, quality traits (normal seedling and dead seed) and reflectance data at 940 nm for three seedlots with different vigor levels (low vigor = L1 [black circles], high vigor = L2 [red triangles], and medium vigor = L3 [green crosses]); abnormal seedling variable did not appear in the model because it is collinear with dead seed (n = 4 repetitions of 25 seeds)

Radiographic images obtained from all seedlots were separated into three different classes (Fig. 6a) based on seed tissue integrity and seed performance in the germination test. Soft tissues are associated with deteriorated tissues and they absorb the X-ray beam less as it passed through the tissue, therefore, these areas appear dark in the radiographic images. Meanwhile, regions with high gray intensity indicate greater penetration of X-rays directly associated with higher tissue density (healthy tissues). In class 1, seeds were completely filled or with slight empty spaces (≤ 1.23 mm) between the endosperm and the seed coat, and these seeds mainly generated normal seedlings. Seeds in class 2 showed large empty spaces (> 1.24 mm) between the endosperm and the seed coat or deteriorated tissues without reaching the embryonic axis, and they produced mostly abnormal seedlings. Finally, class 3 included seeds with deteriorated tissues reaching the embryonic axis, malformed and empty seeds, which were directly associated with dead seeds.

The CDA method (Fig. 6b) showed that Lot 1 was positively correlated with class 2 and dead seeds. The abnormal seedling variable did not appear in the model because it was collinear with dead seeds. Seeds of Lot 2 were positively correlated with X-ray images in class 1 and normal seedlings, and it was completely opposite to Lots 1 and 3. Reflectance data at 940 nm distinguished Lot 3 from Lots 1 and 2, with higher amount of seeds in class 3 and dead seeds (*F* = 10.22, df = 2, 12, *P* = 0.0014).

### Validation

**Fig. 7:**
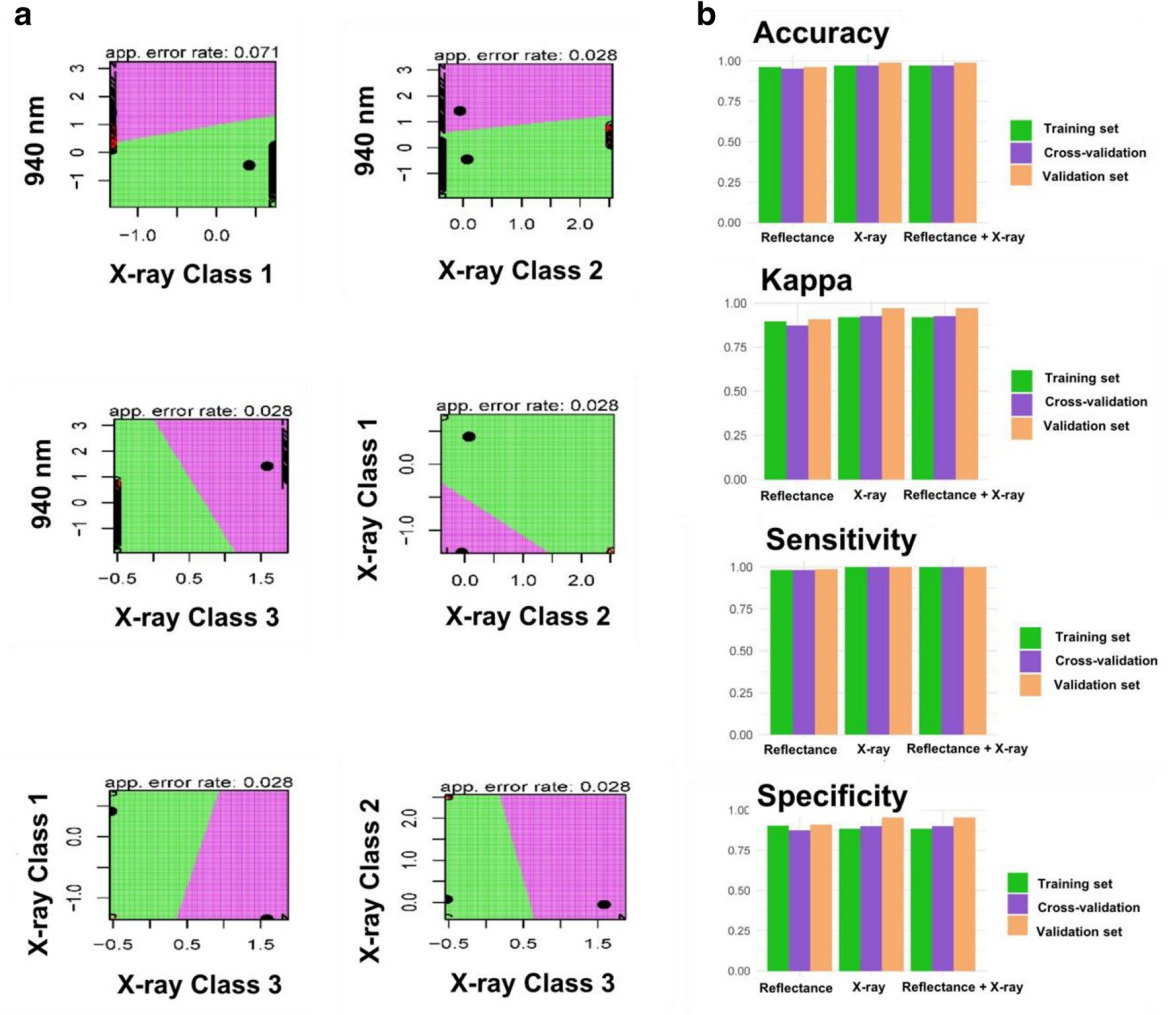
Quality prediction of *Jatropha curcas* seeds based on reflectance at 940 nm and X-ray classes using a Linear Discriminant Analysis (LDA). **a** Plots partitioning two variables and apparent error rates; colored regions delineate each classification area and the observations (spots) within a region is predicted to be from a specific class. **b** Metrics used to validate the models based on accuracy, kappa, sensitivity and specificity

We created models to validate the proposed approach using a Linear Discriminant Analysis (LDA) algorithm. These models were developed using reflectance data at 940 nm and X-ray classes, individually or in combination (Fig. 7). All models showed good performance in the validation set, with predictive accuracy of 0.96, 0.98 and 0.98 using reflectance, X-ray and the combination of reflectance + X-ray data, respectively.

## Discussion

In seed industry, the development of non-destructive methods for quality control and screening is still a challenge. High-quality seeds are relevant for the entire seed business, which include breeders, producers, traders, variety registration agencies and distributers. Germination and vigor tests are the methods most widely employed for seed quality assessment [17], and the greater seed germination and vigor the better seedling performance in the field. Germination tests determine the ability to produce normal seedlings under favorable field condition, and vigor tests estimate the potential for rapid and uniform emergence of normal seedlings under a wide range of field conditions [17].

In this study, we present an approach based on two advanced imaging techniques for assessing seed quality using multispectral and X-ray data. Our studies included the combination of these techniques because multispectral imaging has a focus on seed surface profile, which may be negatively affected without reaching important internal regions of seeds. An oilseed plant was used as a model. Initially, seed performance of three lots was tested using conventional analytical methods, which showed that the three seedlots significantly differed in terms of germination capacity and vigor. Results also showed differences in crude fat content, but not for crude protein. A PCA method was applied to the multispectral data, which revealed the most meaningful wavelengths to distinguish the seedlots (780, 850, 880, 940 and 970 nm). The group identified as high-vigor seeds (Lot 2) had the highest crude fat content and the lowest reflectance spectra for both ventral and dorsal seed surfaces. In addition, this group presented more radiographic images in class 1, i.e., with tissues completely filling the seed or slight empty space between the endosperm and the seed coat, which was positively correlated with normal seedlings in the germination test.

**Fig. 8:**
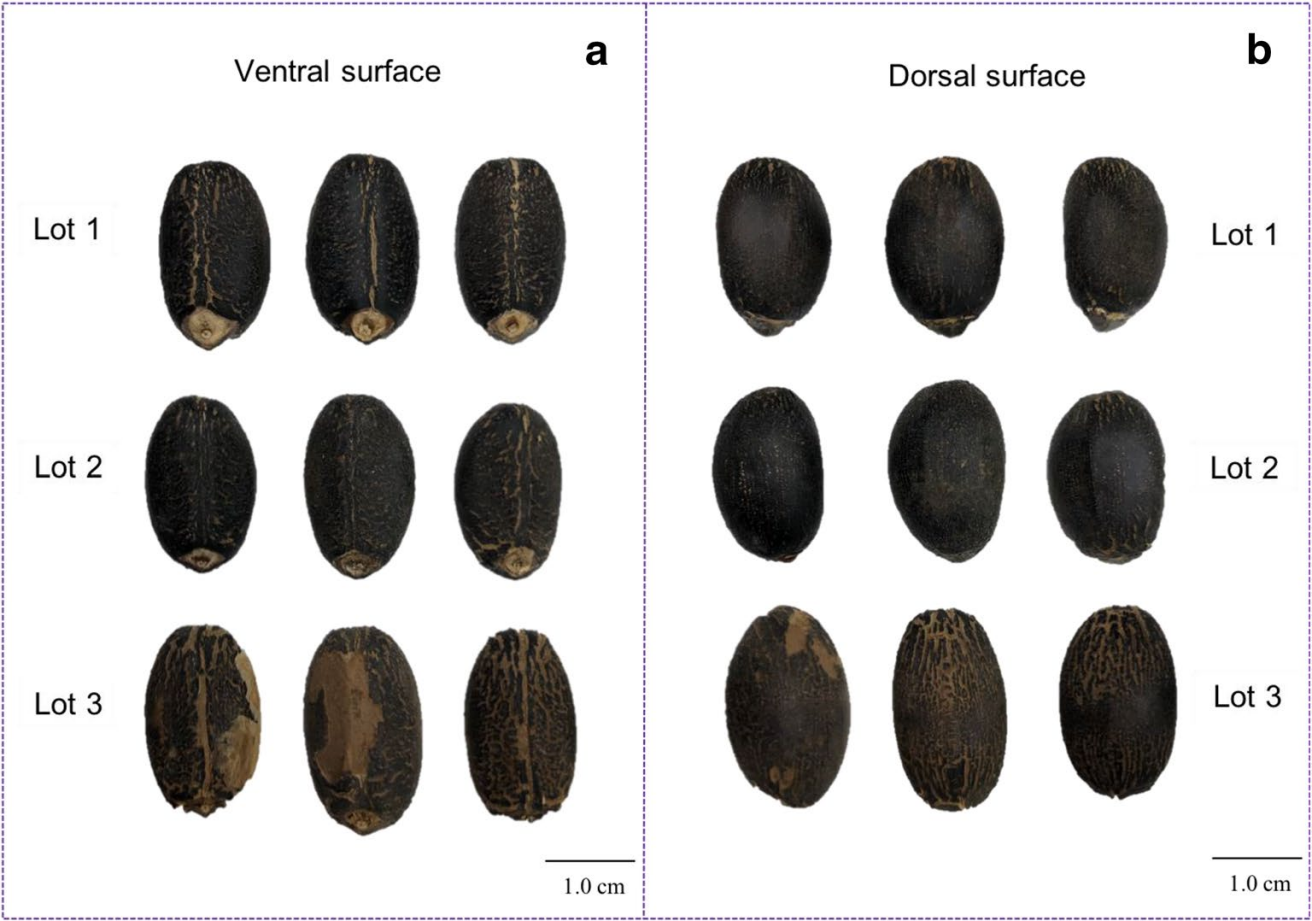
Overview of ventral (**a**) and dorsal (**b**) surface of three seedlots (Lots 1, 2 and 3) of *Jatropha curcas*

There are several reasons to explain why high-vigor seeds have lower reflectance values. When light strikes an object, its rays can be reflected, transmitted, scatted, or absorbed. Reflectance properties of an object depend on its physical and chemical states [18]. In the NIR region, distinctive spectral data correspond to energy absorption of functional groups containing a hydrogen atom (combination of C-H, N-H and O-H) [19]. The 970 nm wavelength is associated with water [20], and there is evidence of absorption peak of fatty tissues at 890 and 940 nm [20, 21]. As a consequence of this, fatty tissues are less reflective as shown in seeds of Lot 2; however, the reflectance data also depend on the color in which the brightest regions are most reflective [22]. For example, there were lighter colored tissues for the ventral and dorsal seed surface in Lot 3 (medium vigor) (Fig. 8) that may have increased the reflectance intensity, i.e., although Lot 3 showed a slight tendency towards higher crude fat content than Lot 1 (low vigor) (Fig. 1), Lot 3 exhibited the highest reflectance (Fig. 2).

High fat content in seeds has been associated with rapid availability of energy and increased mobilization of reserves during germination [1, 23]; therefore, the high percentage of crude fat in Lot 2 may explain the rapid emergence of its seedlings. In previous studies, fat was the main molecule mobilized during germination of *Cereus jamacaru* seeds [24]. Hence, seed oil content is an important seed quality parameter in oilseed species [7].

It has been demonstrated that unhealthy tissues or non-viable seeds are not good absorbers of NIR energy [25–28]. This was also verified in Lots 1 and 3, which showed a higher proportion of deteriorated tissues in the radiographic images (seeds in classes 2 and 3), higher reflectance values in the NIR region and more dead seeds in the germination test. A reflectance trend toward higher values in the NIR spectra was also found in non-viable seeds of *Brassica oleracea* [29] and *Ricinus cummunis* [26]. Healthier tissues are metabolically more active; therefore, they absorb more light energy and reflect less.

There was a direct relationship between jatropha seeds with deteriorated tissues in radiographic images and abnormal seedlings or dead seeds in the germination test. But, dead seeds occurred mainly when deteriorated tissues were reaching the embryonic axis (X-ray images in class 3). Lower grayscale in the X-ray images are strongly related to lower physical integrity and less stored reserves, including protein, carbohydrates and fats [30].

All lots used in this study showed a mixture of normal seedlings, abnormal seedlings and dead seeds, therefore, although Lots 1 and 2 had the most extreme quality difference, these lots showed some seeds with similar spectral signature that overlapped in Can 1 (Fig. 4). A similar behavior was also found in the separation of early germinated seeds from medium germination and dead seeds in cowpea using an LDA model [31].

Results obtained in this study would not be achieved using RGB images because they are limited to the visible light spectrum. Moreover, other traditional reflectance techniques such as NIR spectroscopy measure only a relatively small area of a specimen (spot measurement where the sensor is located), so they do not provide spatial information that is important for many seed inspection applications. NIR spectroscopy can be successful used when the attribute measured related to seed quality (e.g. disease, damage and phenolic compound) is located in specific regions of seeds [11, 32], but this method requires spectral pre-processing methods to remove irrelevant information and improve the performance of calibration models [11, 28, 33, 34]. On the other hand, NIR spectral imaging has provided spatial and spectral information of samples using different wavelengths to obtain rapid and accurate measurements of uniform and non-homogeneous samples. It has been used to predict seed health status [35], discrimination of seeds at different maturation stages [36] or with mechanical damage [37].

Different from multispectral imaging that generates reflectance images, X-ray technique produces transmittance images resulting from short wavelengths that can penetrate seed tissues [9, 38]. X-ray tests are well known in detecting internal seed defects, which contributes to predict problems in the future such as physiological abnormalities during imbibition, germination and seedling development [8, 9, 39, 40]. It has been successfully used to identify insect damages in *Triticum aestivum* [39, 41] and *Glycine max* seeds [42], desiccation sensitivity of *Quercus rubra* seeds [43], and mechanical damage in *Archontophoenix cunninghamii* seeds [44].

Our findings indicate that multispectral and X-ray imaging techniques have the potential to objectively predict seed quality, with high accuracy (0.96–0.98). Jatropha seed is known for its short-term storage [45], therefore, rapid, simple and accurate methods can help producers and distributors to ensure seed quality. However, in-depth studies with a larger number of seedlots from different regions and crop-year are still needed to strengthen the methodologies for applying multispectral and X-ray imaging in the quality inspection of jatropha seeds or other important oilseeds.

## Conclusion

Seed quality is mainly monitored by destructive, laborious and time-consuming methods that require specialized analysts. In this research, we demonstrated two non-destructive techniques for seed quality characterization based on multispectral and X-ray images. Three different classes of jatropha seeds were tested. We proposed an approach using X-ray images to investigate internal aspects of seeds, such as deteriorated tissues in the embryonic axis and endosperm, considering the fact that seed surface can be negatively affected without reaching important internal regions of seeds. We compared multispectral and X-ray data with analytical methods traditionally used to evaluate seed performance, including germination tests, electrical conductivity and seedling emergence. Our results demonstrated that multispectral and X-ray images have a strong relationship with seed physiological potential*.* Reflectance data at 940 nm and X-ray data, individually or combined, showed above 0.96 accuracy to predict quality traits such as normal seedlings, abnormal seedlings and dead seeds. Multispectral and X-ray imaging could be used for rapid, sustainable and non-destructive evaluation of seed quality in the future, overcoming intrinsic subjectivities of seed testing.

## Methods

### Seed material

Jatropha plants have a great variation in the fruit ripening time, with the same plant showing fruits at different stages of ripeness [46]. In this study, changes of pericarp color were used as indicators of ripening, and all fruits were collected in the ‘brown dry’ maturity stage [47]. Three different seedlots (Lots 1, 2 and 3) were investigated. After the harvest, fruits were kept at room temperature for one week. Then, seeds were extracted manually from the fruits and each seedlot was homogenized and evaluated for moisture content (fresh weight basis) which ranged from 11.3 to 11.8%. All seedlots were packed in Kraft paper bags and stored at 20 ºC and 40% RU during the experimental period. In this condition, the seed water content was reduced, varying between 6.5 and 6.6%. Traditional tests were performed to rank the lots based on germination and vigor.

### Traditional tests to rank lots based on germination and vigor

#### Germination tests

Seeds were sown on paper towel and sand substrates and kept at 30 ºC and a photoperiod of 12 h: ten repetitions of 10 seeds per lot were distributed on paper towels moistened with distilled water (1: 2.5, g: ml), and four replications of 25 seeds per lot were sown in sand (moistened to 60% of its water holding capacity) in plastic trays. The percentage of normal seedlings per lot were recorded at 5 and 10 days after sowing. To calculate the germination rate index–GRI [48], the number of emerged seedlings on paper substrate was monitored daily during 10 days.

#### Electrical conductivity

Four replications of 15 seeds per lot were weighed and maintained for 6 h in containers with 75 mL of distilled water at 25 °C [49]. The electrical conductivity (μS cm^−1^ g^−1^) was measured using a DIGIMED DM-32 conductivity meter.

#### Seedling emergence

Four subsamples of 25 seeds per lot were sown in plastic trays containing sand moistened to 60% of its water holding capacity. Boxes were maintained at room temperature. The percentage of emerged seedlings was determined at 10 days after sowing.

Data from germination tests, electrical conductivity and seedling emergence were analyzed separately by analysis of variance in a completely randomized design and the means compared by the Tukey’s test (*P* < 0.05).

#### Fat and protein content

Proximate chemical composition analysis of the seeds was performed according to the methods of the Association of Official Analytical Chemists [50] for crude fat (AOAC N^o^.4.5.01) and crude protein content (AOAC N^o^.4.2.11). Percent data of crude fat and crude protein content were separately fitted to a linear model with normal distribution for errors, including seedlot as the fixed effect in the linear predictor. Post-hoc contrasts between seedlots were further determined by Tukey test (*P* < 0.05).

### Multispectral imaging

Multispectral images were obtained using a VideometerLab4 (Videometer A/S, Herlev, Denmark) and its software VideometerLab version 3.14.9. This instrument is integrated with a sphere providing homogeneous and diffuse illumination using strobe light-emitting diode (LED) technology. Reflectance images were captured at 19 different wavelengths (365, 405, 430, 450, 470, 490, 515, 540, 570, 590, 630, 645, 660, 690, 780, 850, 880, 940 and 970 nm), combining them into high-resolution multispectral images (40 μm/pixel). Every pixel in the image contains reflectance data, which varies depending on color, texture and chemical composition of the sample.

Ten replications of 10 seeds per lot were placed in 9-cm Petri dishes. Before image acquisition, the individual and automated adjustment of light intensity in each wavelength band was performed to optimize the illumination for the specific type of sample, resulting in an improved signal-to-noise ratio in such a way that the multispectral images captured from different seed classes could be directly comparable. Light setup was adjusted using a representative sample area, then the strobe time of each illumination type was optimized with respect to this area. The auto light assures an optical dynamic range of each band without saturation within the auto light ROI. Subsequently, the instrument was calibrated using three calibration targets: (i) uniform bright disc, (ii) uniform dark disc, and (iii) geometric disc which is black with dots in a rectangular grid.

Multispectral images were captured from both ventral and dorsal seed surface of 10 samples with 10 seeds per lot. The overview of the ventral and dorsal surfaces of the three seedlots is shown in Fig. 8. After successive lighting using 19 LEDs (sequential strobes), multispectral images of a sample (plate with 10 seeds) were captured in a few seconds, requiring no sample preparation. The ROI of each seed was extracted into a Binary Large Object (BLOB) toolbox, a built-in function in VideometerLab software; each BLOB was a representation of one seed. Mean spectra were plotted to show the difference among the three seedlots based on their multispectral signatures. A normalized canonical discriminant analysis (nCDA) algorithm was used as a supervised model based on multispectral image transformation, which allows to minimize the distance to observations within seedlot and to maximize the distance to observations among seedlots.

We applied a PCA method to process the multispectral data using the “FactoMiner” package [51]. A biplot using the first two principal components (PC1 and PC2) was built to select the most meaningful wavelengths to discriminate the seedlots, according to Pearson’ correlation test (*P* < 0.05). Multispectral data corresponding to only meaningful wavelengths, as previously assigned by PCA were used in a CDA model implemented with a “candisc” package [52]. We tested the effect of low, high and medium vigor (i.e., three classes of seed physiological potential) on the multispectral data using a multivariate analysis of variance (MANOVA). The statistical analyses were performed using VideometerLab software and the “free software environment for statistical computing and graphics” R [53].

### X-ray imaging

In total, 100 seeds per lot were radiographed. Seeds were numbered and fixed on an adhesive paper in groups of 10 seeds. Radiographic images were generated using a MultiFocus digital radiography system (Faxitron Bioptics LLC, USA). This system is equipped with a complementary metal–oxide–semiconductor (CMOS) X-ray sensor coupled with an 11 μm focal spot tube and up to 8X geometric magnification and provides as high as 6 μm resolution for seed imaging with a choice of a 48 μm or 24 μm. The built-in advanced Automatic Exposure Control selects the appropriate exposure time and kV settings for each sample.

After X-ray imaging, four repetitions of 25 seeds were sown in sand (moistened to 60% of its water holding capacity) placed in plastic boxes (32.0 × 28.0 × 10.0 cm), kept at 30 ºC and photoperiod of 12 h. At 10 days after sowing, the individual seeds were evaluated for different quality traits: normal seedlings, abnormal seedlings and dead seeds. Next, they were separated into three different classes based on seed performance in the germination test and tissue integrity in the radiographic images. A CDA analysis was implemented by “candisc” package in R [52] to provide the best discrimination among seedlots categories using a dataset derived from X-ray classes, reflectance data at 940 nm and quality traits (normal seedling, abnormal seedling and dead seed).

### Validation

Three models were developed using LDA algorithm. The first model was created using multispectral data at 940 nm. Data obtained from X-ray classes were used to develop the second model. Finally, multispectral and X-ray data were combined to create the third classification model. In total, 300 seeds were used to develop the models. Training was run with 210 seeds (70%), and the remaining 90 seeds (30%) were used for independent validation set. Additionally, fivefold cross-validation was performed using training data. The metrics of accuracy, Cohen's Kappa coefficient, sensitivity and specificity were calculated using a confusion matrix to evaluate the models. Data analysis was performed by R software using the “caret” package [54].

## Data Availability

The datasets used and analyzed during the current study are available from the corresponding author on reasonable request.

## Abbreviations

ANOVA: Analysis of variance
BLOB: Binary large object
CDA: Canonical discriminant analysis
CMOS: Complementary metal–oxide–semiconductor
GRI: Germination rate index
LDA: Linear discriminant analysis
LED: Light-emitting diode
MANOVA: Multivariate analysis of variance
nCDA: Normalized canonical discriminant analysis
NIR: Near infrared
PCA: Principal component analysis
ROIs: Region of interest
RU: Relative humidity
